# Retained Guidewire Penetrating the Spinal Cord: An Unusual Cause of Cervicobrachialgia

**DOI:** 10.7759/cureus.94977

**Published:** 2025-10-20

**Authors:** Simon Bertrand, Simon Landsweerdt, Aurélie Leroux, Julien Flament

**Affiliations:** 1 Department of Cardio-Vascular and Thoracic Surgery, Centre Hospitalier Universitaire (University Hospital Centre) UCL Namur – site Godinne, Yvoir, BEL; 2 Department of Emergency Medicine, Centre Hospitalier Universitaire (University Hospital Centre) UCL Namur – site Godinne, Yvoir, BEL

**Keywords:** arterial catheterization, cervicobrachialgia, peripheral vascular surgery, retained guidewire, spinal cord injury, surgical case report

## Abstract

Retained guidewires are a rare but serious complication of vascular catheterization, most often associated with central venous access. Arterial guidewire retention is exceptionally uncommon, and to our knowledge, no previous case has been documented with migration into the spinal cord. We report the case of a 63-year-old woman who developed progressive cervicobrachialgia due to a retained guidewire extending from the left subclavian artery to the brachial artery, with direct penetration into the cervical spinal cord. The guidewire had been inadvertently left behind during radial artery catheterization and remained undetected for four years. The patient’s initial symptoms were managed conservatively, but recurrence prompted advanced imaging that revealed the intravascular foreign body. She underwent a successful open surgical removal through combined subclavian and brachial approaches, followed by an uneventful recovery and significant neurological improvement. This case highlights a unique and unprecedented complication of arterial guidewire retention, emphasizing the potential for delayed neurological sequelae when recognition is postponed. Thorough clinical evaluation with imaging should be considered in patients with unexplained pain after catheter-based procedures. Early detection, meticulous procedural technique, and timely surgical intervention are crucial for preventing long-term morbidity and achieving favorable outcomes.

## Introduction

Arterial catheterization is a routine procedure performed in various clinical settings, including the intensive care unit, emergency room, and operating room, for continuous hemodynamic monitoring and blood gas sampling [1]. Approximately 8 million and 2.5 million arterial catheters are placed annually in the USA and Europe, respectively [2]. The radial artery is the preferred site for arterial catheterization due to its superficial course and low complication rate [3]. The use of a guidewire allows safe and accurate catheter placement by facilitating arterial access and minimizing the risk of vascular injury or catheter misplacement. The guidewire is removed after a successful catheter insertion [4].

The incidence of complications from arterial catheterization in adult patients typically ranges from 10% to 13%, depending on the placement site. Common complications include local pain, bruising, hematoma, thrombosis, pseudoaneurysm, vasospasm, dissection, arteriovenous fistula, and air or particulate embolism [4]. Ultrasound-guided techniques and strict adherence to sterile procedures can significantly reduce many clinically significant complications, with temporary arterial occlusion being the most common [4-6].

Among these complications, a lost guidewire is the most serious, as it is entirely preventable if the practitioner meticulously performs catheter insertion [7]. Although the literature reports instances of lost and forgotten guidewires, these are almost exclusively associated with central venous catheterization or endovascular therapy. However, no cases report the migration of a guidewire into the spinal cord.

Here, we describe a patient who sustained a penetrating injury to the cervical spinal cord due to a guidewire from an arterial catheterization.

## Case presentation

A 63-year-old female patient underwent an uncomplicated thyroidectomy with cervical lymph node dissection four years ago. Preoperatively, an anesthetist inserted an arterial catheter via the left radial artery. Postoperatively, she experienced transient neck pain, which resolved spontaneously.

Two weeks after the surgery, the patient complained of left arm pain and a mobility deficit. A Doppler ultrasonography revealed arterial thrombosis extending from the radial to the humeral artery. A surgical thrombectomy was initially planned; however, the symptoms spontaneously resolved in the operating room, leading to the cancellation of the procedure. Peripheral arterial flow was controlled by pen-tip doppler and was normal. No further radiological investigations were conducted in this context.

**Figure 1:**
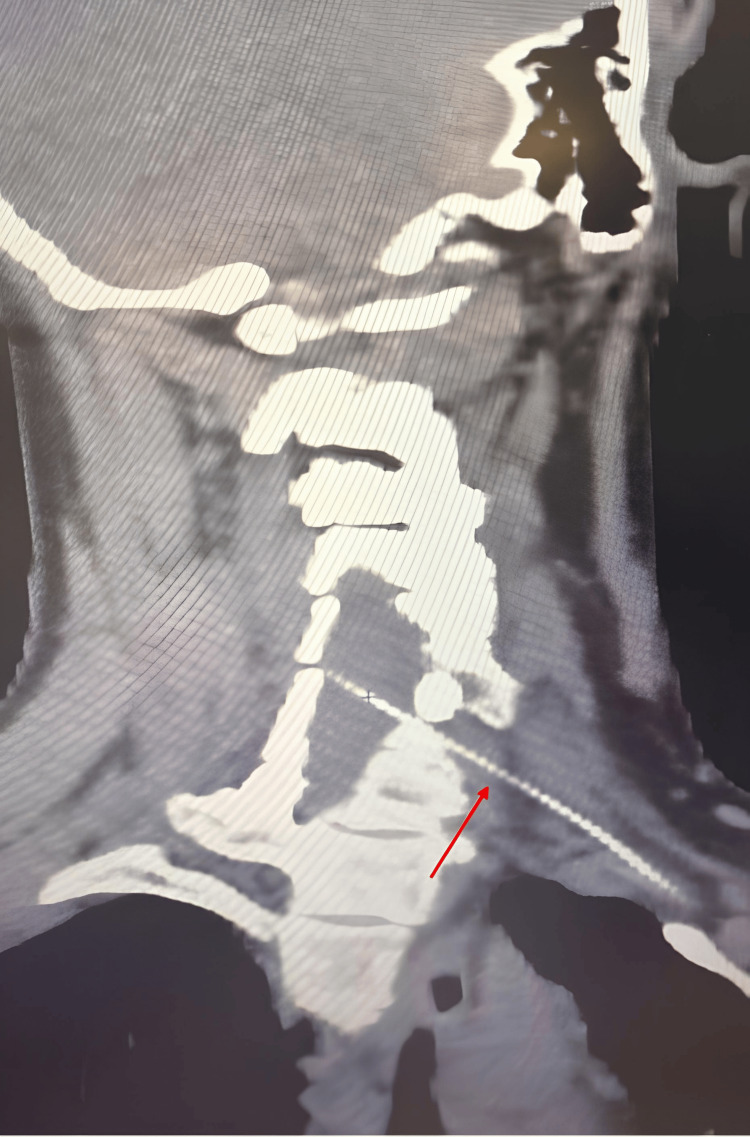
CT scan performed four years after thyroidectomy, showing the foreign body (red arrow) extending from the left subclavian artery toward the spinal cord

**Figure 2:**
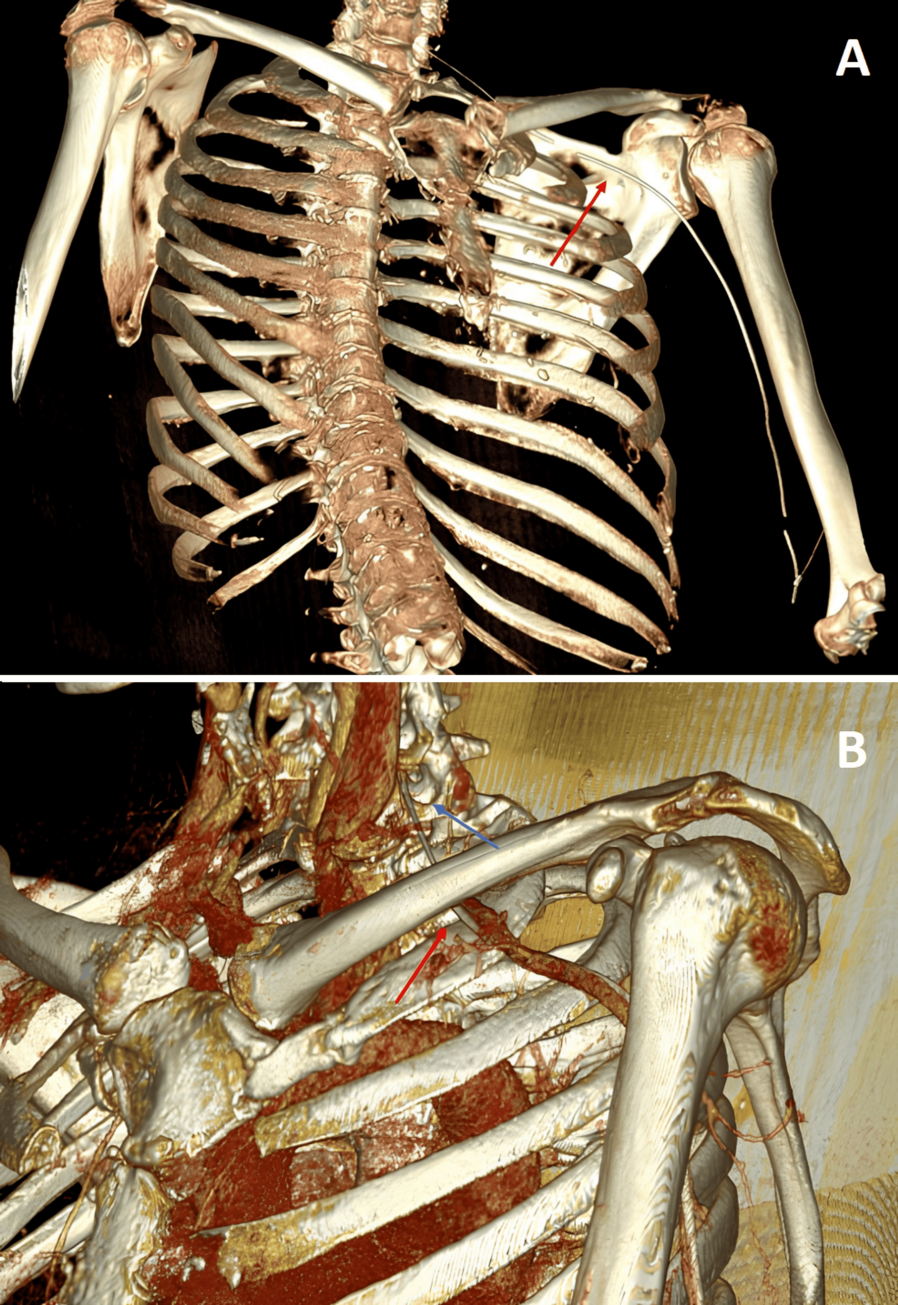
Imaging reconstruction showing the guidewire (red arrow) passing through the intervertebral foramen (blue arrow) (A) Right anterolateral view, and (B), left anterolateral view.

Four years later, the patient was hospitalized in the neurosurgery department for cervicobrachial pain in the right arm without motor or sensory deficits. A CT scan was performed and revealed a 10 cm foreign body extending from the intramedullary C6 to the left thoracic region. She was discharged from the hospital after five days of pain management. Two hours later, she returned to our emergency department with recurrent symptoms. A new CT scan and echography confirmed the presence of the intravascular foreign body, extending from the left subclavian to the left brachial artery (Figures 1, 2).

The artery was not occluded.

**Figure 3:**
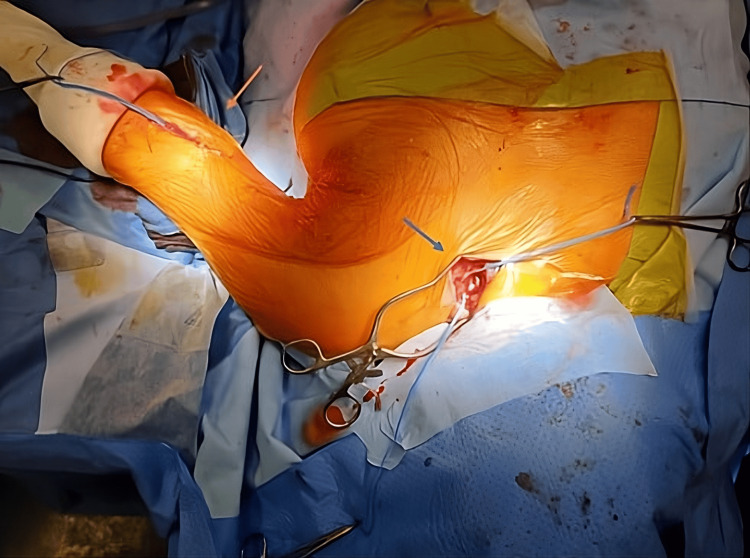
Surgical exposure via open subclavian (blue arrow) and brachial (orange arrow) access to allow safe extraction of the guidewire

She was admitted to our vascular surgery unit for the removal of the foreign body by open subclavian and brachial access (Figure 3).

**Figure 4:**
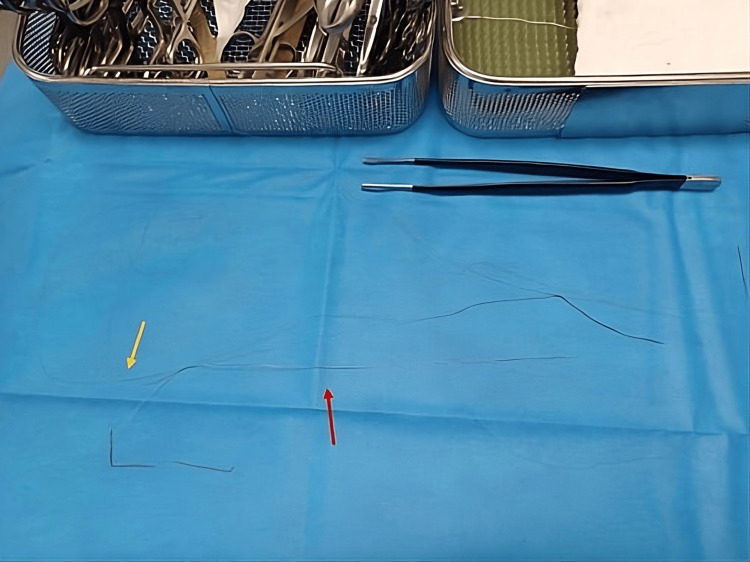
The guidewire extracted from the artery The core of the wire (red arrow) is separated from the outer sheath (yellow arrow) upon removal.

The retrieved object was identified as a guidewire that had dissected through the artery walls and penetrated the spinal cord (Figure 4).

The postoperative course was uncomplicated. Pain and dysesthesia were initially managed with Lyrica (pregabalin) 75 mg twice daily, supplemented with Dafalgan (paracetamol) and tramadol as needed. One month after surgery, the cervicobrachial pain had significantly decreased, and the patient subsequently discontinued all analgesic medication on her own. By seven months after the operation, the pain had completely resolved; however, she continued to experience persistent dysesthesia.

## Discussion

Although percutaneous catheterization of central veins or arterial lines is a common procedure, it demands advanced surgical skills, close supervision, and careful attention to detail to avoid adverse effects. The loss of a guidewire or catheter, although preventable, is an occasional complication documented in the literature. In some instances, these cases were not immediately recognized after catheter insertion and led to delayed complications [8].

For acute penetrating injuries or suspected metallic foreign bodies, initial imaging modalities include radiographs and CT scans. Although this has been debated, MR imaging has been proven safe even in the presence of intraspinous metallic debris and remains crucial for detecting foreign bodies and complications such as epidural hematomas or cord contusions [9-11].

Foreign bodies typically enter the spinal canal through the interlaminar space or intervertebral foramen. The delayed onset of neurological symptoms is often due to retained fragments within the spinal canal, which can transfix the spinal cord, causing progressive damage through tethering. Even after surgical removal, residual fibrosis can persist [12].

When a lost guidewire is identified and retrieval is attempted, two primary methods are available: open surgery and the percutaneous (endovascular) method. The traditional surgical approach involves exploring the blood vessels at the site indicated by radiographic imaging. However, minimally invasive techniques are usually preferred before resorting to major surgery, with the snare being the primary tool for endovascular retrieval. In some cases, endovascular retrieval may prove difficult, necessitating immediate surgical intervention [8,13]. 

In this case, the guidewire had been lost for four years and had migrated into the spinal cord, causing symptoms. This warranted the decision to opt for open surgery, which successfully retrieved the guidewire with minimal morbidity.

## Conclusions

Complications from missed and lost guidewires are relatively rare in clinical practice, requiring practitioners to remain vigilant for related symptoms. Immediate removal of detected guidewires in the surgical field can prevent serious sequelae. While radiological intervention is often the preferred treatment, surgical procedures may also be necessary. Given the rarity of lost guidewires, predicting clinical outcomes post-retrieval is challenging; however, most cases resolve without incident.

In this case, a guidewire lost in the left radial artery migrated and penetrated the spinal cord, causing recurrent cervicobrachial pain. Clinically, the case is notable for two reasons: the guidewire remained undetected for four years, and it eventually penetrated from the brachial artery into the spinal cord. To the best of our knowledge, this is the first report of cervicobrachialgia caused by a missed guidewire. Although rare, the potential complications of lost guidewires must not be overlooked.
